# Protection Against Marburg Virus Using a Recombinant VSV-Vaccine Depends on T and B Cell Activation

**DOI:** 10.3389/fimmu.2018.03071

**Published:** 2019-01-22

**Authors:** Andrea Marzi, Andrea R. Menicucci, Flora Engelmann, Julie Callison, Eva J. Horne, Friederike Feldmann, Allen Jankeel, Heinz Feldmann, Ilhem Messaoudi

**Affiliations:** ^1^Laboratory of Virology, Division of Intramural Research, National Institute of Allergy and Infectious Diseases, National Institutes of Health, Hamilton, MT, United States; ^2^Department of Molecular Biology and Biochemistry, University of California, Irvine, Irvine, CA, United States; ^3^Department of Cell Molecular Biology, Northwestern University, Evanston, IL, United States; ^4^Rocky Mountain Veterinary Branch, Division of Intramural Research, National Institute of Allergy and Infectious Diseases, National Institutes of Health, Hamilton, MT, United States

**Keywords:** filovirus, MARV Angola, VSV-MARV, nonhuman primate model, macaque

## Abstract

Marburg virus (MARV) is the causative agent of hemorrhagic fever outbreaks with high case fatality rates. Closely related to Ebola virus, MARV is a filamentous virus with a negative-sense, single-stranded RNA genome. Although extensive studies on filovirus countermeasures have been conducted, there are no licensed treatments against MARV infections. An experimental vaccine based on the recombinant vesicular stomatitis virus (VSV) expressing the MARV-Musoke glycoprotein demonstrated complete protection when a single dose was administered 28 days and up to 14 months prior to MARV challenge. Here, we analyzed the protective efficacy of an updated vaccine expressing the MARV-Angola glycoprotein (VSV-MARV). A single dose of VSV-MARV given 5 weeks before challenge provided uniform protection with no detectable viremia. The vaccine induced B and T cell proliferation and, importantly, antigen-specific IgG production. Transcriptomic signatures confirm these findings and suggest innate immunity engendered by VSV-MARV may direct the development of protective humoral immunity.

## Introduction

Marburg virus (MARV) is a member of the *Filoviridae*, a family of non-segmented, negative-stranded RNA viruses that cause severe hemorrhagic fever with case fatality rates (CFR) ranging from 23 to 90% in humans and 100% lethality in nonhuman primates (NHPs) following experimental infection (1). The first MARV outbreaks occurred simultaneously in Germany and Serbia in 1967 where they resulted in 31 cases and 7 deaths (2–5). Studies traced the outbreaks to a shipment of infected African green (Vervet) monkeys imported from Uganda (2, 3). Since 1967, several outbreaks and sporadic cases have been reported in Angola, The Democratic Republic of Congo (DRC), Kenya, South Africa and Uganda, with the largest outbreaks occurring in 1998–2000 in the DRC (154 cases, 83% CFR) and in 2004–2005 in Angola [374 cases, 88% CFR (6)]; in addition, single human MARV cases were imported into the Netherlands and the USA (1).

Marburg hemorrhagic fever (MHF) is similar to the disease caused by Ebola virus (EBOV) and is characterized by fever, an excessive inflammatory response, coagulation abnormalities, and vascular hemorrhaging (1). Analysis of tissue samples from infected NHPs demonstrated that, as previously described for EBOV, monocytes and dendritic cells (DC) in lymphoid tissues as well as Kupffer cells in the liver are early targets of MARV infection (7). Disseminated intravascular coagulation was noted at the late stages of disease as evidenced by increased levels of D-dimers and fibrin deposition in tissues, albeit with reduced severity compared to EBOV infection in NHPs (7).

There are currently no licensed vaccines or therapeutics available against MHF (8). However, several vaccine platforms have shown potential to protect NHPs from MARV infection including DNA vectors, virus like particles (VLPs), recombinant adenovirus vectors (Ad) and recombinant vesicular stomatitis virus (VSV) (9). The VSV-MARV vaccine platform, where the VSV glycoprotein (G) is replaced with the MARV glycoprotein (GP), is the only platform that has shown efficacy both as a preventative vaccine and as a post-exposure treatment (10). Specifically, a single intramuscular (i.m.) injection of VSV-MARV induced a strong humoral immune response and provided complete protection against lethal MARV challenge 28 days later in cynomolgus macaques (11, 12). This vaccine also provides complete protection up to 14 months with circulating anti-MARV GP IgG titers detected over a year following immunization (13). Lastly, complete protection was achieved when animals were treated with VSV-MARV 20–30 min after challenge (14), 5/6 animals were protected when given the vaccine 24 h after challenge (15), and 2/6 animals were protected when treatment started 48 h after infection (15). Similar to the VSV-EBOV vaccine, which was previously evaluated during the 2013–16 West African EBOV epidemic (16) and is currently being used in clinical trials in the 2018 EBOV outbreak in the Democratic Republic of the Congo, the VSV-MARV is attenuated compared to wild-type VSV as evidenced by the lack of neurovirulence in NHPs that were intra-thalamically injected with VSV-MARV or VSV-EBOV (17).

The mechanisms by which the VSV-MARV vaccine provides protection are poorly understood. In this study, we sought to address this knowledge gap by using both immunological assays as well as RNA-Seq to characterize the host immune response in cynomolgus macaques vaccinated with 1 × 10^7^ plaque forming units (pfu) of VSV expressing the MARV-Angola GP 35 days before challenge. We further determined gene expression changes in negative control and vaccinated animals after challenge with MARV-Angola. Data presented herein show that although VSV-MARV vaccination induces T and B cell proliferation, it appears that mainly antibody responses were significant for protection. From a transcriptional standpoint, VSV-MARV vaccination induced large gene expression changes 7 days post vaccination (DPV) which return to baseline levels 14 days later. Differentially expressed genes enriched to gene ontology terms associated with innate immunity and B cell immunity. Following MARV challenge, no gene expression changes were detected in the protected vaccinated animals while negative control animals displayed transcriptional changes associated with viral hemorrhagic fever.

## Materials and Methods

### Ethics Statement

All infectious work with MARV was performed by using standard operating procedures (SOPs) approved by the Rocky Mountain Laboratories (RML) Institutional Biosafety Committee (IBC) in the maximum containment laboratory at the RML, Division of Intramural Research, National Institute of Allergy and Infectious Diseases, National Institutes of Health. Animal work was performed in strict accordance with the recommendations described in the Guide for the Care and Use of Laboratory Animals of the National Institute of Health, the Office of Animal Welfare and the United States Department of Agriculture and was approved by the RML Animal Care and Use Committee (ACUC). Procedures were conducted in animals anesthetized with ketamine by trained personnel under the supervision of veterinary staff. All efforts were made to ameliorate animal welfare and minimize animal suffering in accordance with the Weatherall report on the use of nonhuman primates in research (https://royalsociety.org/policy/publications/2006/weatherall-report/). Animals were housed in adjoining individual primate cages that enabled social interactions, under controlled conditions of humidity, temperature, and light (12 h light:12 h dark cycles). Food and water were available *ad libitum*. Animals were monitored and fed commercial monkey chow, treats, and fruit at least twice a day by trained personnel. Environmental enrichment consisted of commercial toys, music, and video. Endpoint criteria, specified by the RML ACUC-approved clinical score parameters, were used to determine when animals were humanely euthanized.

### Vaccines and Challenge Virus

The VSV-MARV expressing MARV-Angola GP and VSV-EBOV expressing EBOV-Mayinga GP vaccines were propagated, titered and stored as described previously (18). All animals were i.m. vaccinated with 1 × 10^7^ pfu VSV-MARV or VSV-EBOV (negative control group) in 1 ml into the caudal thighs 35 days prior to lethal MARV challenge.

MARV-Angola (passage 2) (19) was propagated on Vero E6 cells, titered via focus-forming unit assay using a MARV GP-specific antibody (kindly provided by Stephan Becker, Philipps University Marburg, Germany) on Vero E6 cells and stored in liquid nitrogen. On day 0, all animals were infected i.m. with 1,000 pfu in 1 ml into the caudal thighs.

### Macaque Study Design

A total of 10 cynomolgus macaques (*Macaca fascularis*), 1 male and 9 female animals, 6–8 years of age and 3–6 kg in weight, were used in this study. The study was not blinded and macaques were randomly divided into 2 study groups. The 6 study animals (5 female, 1 male) were immunized i.m. with 1 × 10^7^ pfu VSV-MARV. The 4 female control animals received the same dose of VSV-EBOV via the same route. All animals were challenged i.m. on day 0 with a lethal dose of 1,000 pfu MARV (confirmed by back-titration). Physical examinations and blood draws were performed on days −35, −28, −21, −14, −7, 0, 4, 7, 14, 21, 28, 35, and 42 as well as at the time of euthanasia. The animals were observed at least twice daily for clinical signs of disease according to an IACUC approved scoring sheet. Due to limited cell numbers, different subsets of animals were used for immunological assays and RNA-Seq analysis.

### Hematology and Serum Chemistries

The total white blood cell, lymphocyte and platelet counts were determined from EDTA blood with the IDEXX ProCyte DX analyzer (IDEXX Laboratories, Westbrook, ME). Serum biochemistry (including AST) was analyzed using the Piccolo Xpress Chemistry Analyzer and Piccolo General Chemistry 13 Panel discs (Abaxis, Union City, CA).

### Virus Loads

For determination of virus loads in macaque blood samples, Vero E6 cells (mycoplasma negative) were seeded in 48-well plates the day before titration. Whole blood samples were thawed and 10-fold serial dilutions were prepared. Media was removed from cells and triplicate wells were inoculated with each dilution. After 1 h, DMEM supplemented with 2% FBS, penicillin/streptomycin and L-glutamine was added and cells were incubated at 37°C. Cells were monitored for cytopathic effect (CPE) and 50% tissue culture infectious dose (TCID_50_) was calculated for each sample employing the Reed and Muench method.

### Flow Cytometry Analysis

PBMCs were surface stained with antibodies against CD8-beta, CD4, CD28, and CD95 to delineate the naïve (CD28+CD95–), central memory (CD29+CD95+), and effector memory (CD28–CD95+) T cell subsets (Biolegend, San Diego, CA). PBMCs were also surface stained with antibodies against CD20, IgD, and CD27 to delineate the naïve (IgD+CD27–), MZ-like (IgD+CD27+), and memory (IgD–CD27+) B cell subsets (Biolegend). Cells were fixed and permeabilized according to manufacturer's recommendations before the addition of a Ki67-specific antibody, which is a cellular marker for proliferation (Biolegend). The samples were analyzed using the LSRII instrument (Becton Dickinson, Franklin Lanes, NJ) and FlowJo software (Tree Star, Ashland, OR).

### Assessment of Humoral Immune Response

Antibody titers directed against MARV GP were measured by ELISA using plates coated with the recombinant protein MARV-Angola GPΔTM (IBT Bioservices, Rockville, MD). Post-challenge NHP sera were inactivate by gamma irradiation (5 Mrad) and removed from the maximum containment laboratory according to RML SOPs approved by the local IBC. ELISA was performed and titers were calculated as previously described (20).

### Assessment of MARV-Specific T Cell Response

Enzyme-linked immunosorbent spot (ELISPOT) assay was carried out as previously described (10). Briefly, 96-well nitrocellulose-bottomed plates were pre-coated with recombinant anti-rhesus IFNγ mAb (MABTECH, Nacka Strand, Sweden). The plates were blocked with RPMI supplemented with 10% (vol/vol) FBS for 30 min at room temperature. A total of 2 × 10^5^ PBMCs/well was stimulated in triplicates with an overlapping peptide library for MARV-GP (15 mers, 10 aa overlap). In the negative control wells, cells were stimulated with DMSO, and in the positive control wells cells were stimulated with 1 μg/mL of PMA/ionomycin (Sigma-Aldrich, St. Louis, MO). After incubation at 37°C for 18 h, the plates were washed with PBS. Biotinylated anti-human IFNγ mAb (MABTECH) at a concentration of 1 μg/mL was added to all wells and the plates were incubated for 1 h at room temperature. Following washing with PBS, BCIP-NBT-plus substrate (MABTECH) was added, and the plates were allowed to develop in the dark for 5–15 min until spots appeared. Color development was stopped by washing with tap water. After drying, the number of GP-specific IFNγ-secreting spot forming cells were counted in the EliSpot reader ELR06 (AID GmbH, Strassberg, Germany) using AID EliSpot software.

### Plasma Cytokine Levels

Post-challenge NHP sera were inactivated by gamma irradiation (5 Mrad) and removed from the maximum containment laboratory according to RML SOPs approved by the local IBC. Serum samples were then diluted 1:2 in serum matrix for analysis with Milliplex Non-Human Primate Magnetic Bead Panel as per manufacturer's instructions (Millipore, Burlington, MA). Concentrations for IL-1β, IL-6, IL-1Ra, MIP-1α, TNFα, IFN-γ, IL-2, and FGF-β were determined for all samples. Values below the limit of detection of the assay were assigned a value one-half that of the lowest value recorded in that assay.

### Library Generation and Sequencing

RNA was isolated from whole blood using the QIAmp Viral RNA Kit (Qiagen, Valencia, CA). RNA concentration and integrity were determined using an Agilent 2,100 Bioanalyzer (Agilent Technologies, Santa Clara, CA). Ribosomal RNA (rRNA) was depleted and libraries were constructed using the TruSeq Stranded Total RNA LT-LS kit (Illumina, San Diego, CA). First, rRNA-depleted RNA was fragmented and converted to double stranded cDNA. Adapters were ligated and the ~300 base pair (bp) long fragments were then amplified by PCR and selected by size exclusion. Each library was prepared with a unique indexed primer for multiplexing. In order to ensure proper sizing, quantitation, and quality prior to sequencing, libraries were analyzed on the Agilent 2,100 Bioanalyzer. Multiplexed libraries were subjected to single-end 75 bp sequencing using the Illumina NextSeq500 platform. Sequence data are available at NCBI BioProject accession number PRJNA508964.

### Bioinformatic Analysis

Data analysis was performed with the RNA-Seq workflow module of the systemPipeR package available on Bioconductor (21). RNA-Seq reads were demultiplexed, quality filtered and trimmed using Trim Galore with an average phred score cutoff of 30 and minimum length of 75 bp. Quality reports were generated with the FastQC function. *Macaca fascicularis* genome sequence (Macaca_fascicularis.Macaca_fascicularis_5.0.dna.toplevel.fa) and the annotation file from Ensembl (Macaca_fascicularis_5.0.94.gtf) were used. In order to determine the level of viral transcription at different time points, the Marburg virus genome (Marburg virus/H.sapiens-tc/AGO) from Virus Pathogen Resource was adjoined to the *Macaca fascicularis* reference. RNA-Seq reads were mapped with the alignment suite Bowtie2/Tophat2 against a reference genome containing both *Macaca fascicularis* and MARV genome sequences. Raw expression values in the form of gene-level read counts were generated with the *summarizeOverlaps* function, counting only the reads overlapping exonic regions of genes, and discarding reads mapping to ambiguous regions of exons from overlapping genes. Normalization and statistical analysis of differentially expressed genes (DEGs) was performed using the *edgeR* package. RNA-sequencing data presented in this article were submitted to the National Center for Biotechnology Information Sequence Read Archive (Accession number pending). Aligned counts for each gene were normalized by correcting for differences in sequencing depth (divide read counts by 1,000,000) and for differences in gene length (in kilobases) in order to obtain reads per kilobase of transcript per million mapped reads (RPKM). Host DEGs were defined as those with a fold change ≥ 2 and a false discovery rate (FDR) corrected *p* ≤ 0.05 relative to baseline pre-vaccination or pre-challenge timepoints. Only protein coding genes with human homologs and an average of 5 reads per kilobase of transcript per million mapped reads (RPKM) were included for further analysis. Reads mapping to the MARV genome were also normalized as RPKM. Heatmaps and venn diagrams were generated using R packages gplot and VennDiagram. Network images were generated using MetaCore^TM^ (Thomson Reuters, New York, NY).

### Functional Enrichment

Functional enrichment of these genes was done to identify clusters of genes mapping to specific biological pathways, specifically gene ontology (GO) terms using MetaCore^TM^.

### Statistical Analysis

Longitudinal changes of clinical parameters, immune cell frequencies and cytokine levels were carried out using one-way repeated measures ANOVA test followed by Dunnett's multiple comparison post-test to determine differences. Statistical significance for all comparisons was determined at the alpha level of 0.05.

## Results

### Immunization With VSV-MARV Induces a Robust Antibody Response

**Figure 1:**
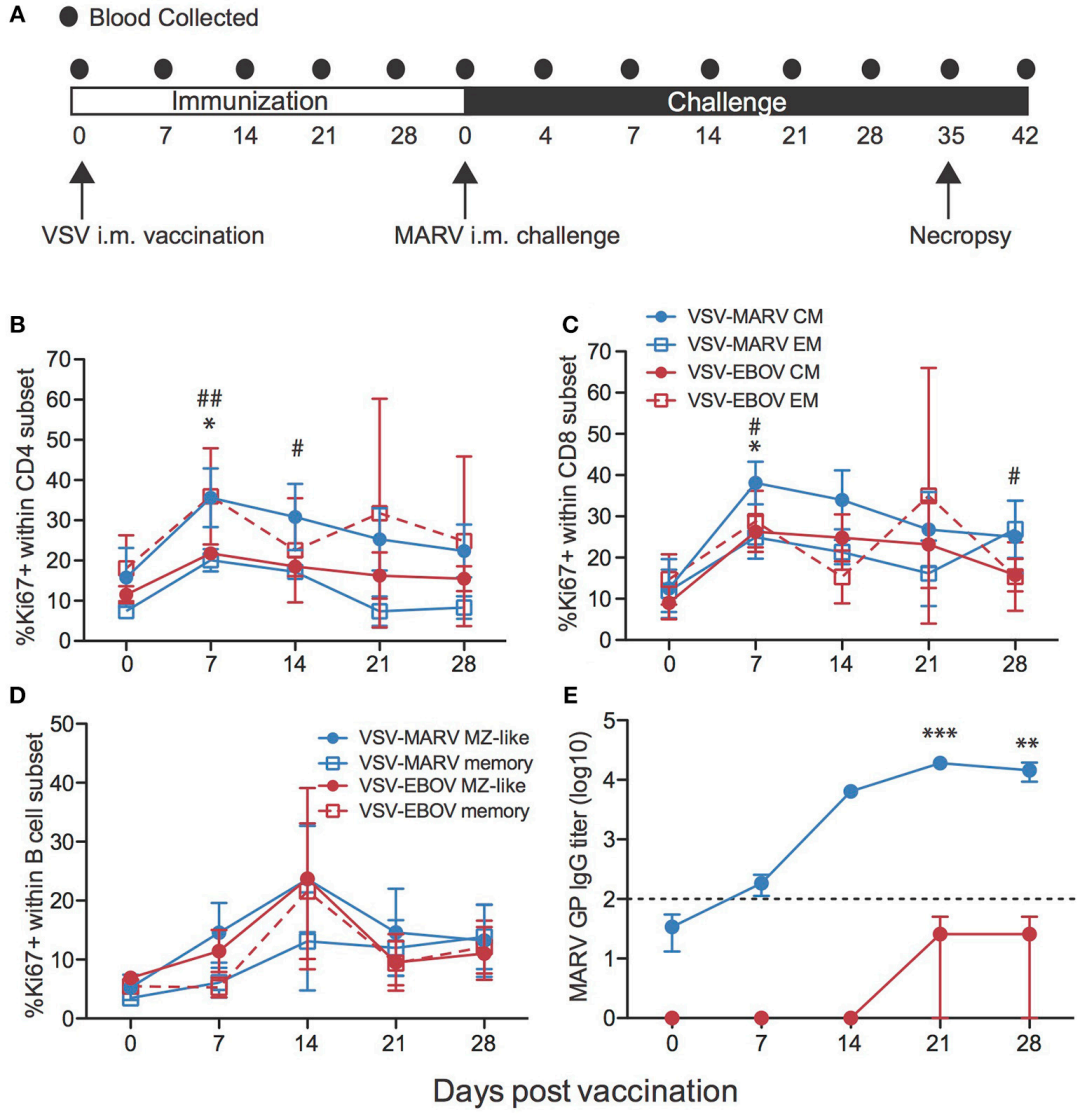
Immune response to VSV-MARV vaccination. **(A)** Time line detailing blood sample collection during vaccine and challenge phases of the study. **(B,C)** Proliferation was determined by measuring changes in the frequency of Ki67^+^ cells within central (CM) and effector (EM) memory cells within CD4 **(B)** and CD8 **(C)** T cell subsets for each group (VSV-EBOV *n* = 2; VSV-MARV *n* = 3). **(D)** Frequency of Ki67^+^ cells within MZ-like B cells and memory B cells for each group. **(E)** MARV GP-specific IgG endpoint titers were measured by ELISA (VSV-EBOV *n* = 4; VSV-MARV *n* = 6); line graph represents limit of detection. Longitudinal changes were assessed using one-way repeated measures ANOVA test followed by Dunnett's multiple comparison post-test to determine differences between the time of vaccination (day 0) and subsequent DPV. **(B,C)** ^*^Denotes EM subsets VSV-EBOV vaccinated animals, # denotes CM subsets VSV-MARV vaccinated animals; ^*^,^#^ *p* < 0.05; ^*##*^*p* < 0.01. **(E)** ^*^Denotes IgG titer VSV-MARV vaccinated animals ^**^p < 0.01; ^***^p < 0.001.

VSV-MARV expressing the MARV-Angola GP was used for this study; we generated this vector in order to update the vaccine to express the most recently circulating GP in Africa. This VSV-MARV vaccine shows enhanced *in vitro* replication kinetics compared to the original VSV-MARV vaccine expressing the MARV-Musoke GP (18). To assess immune responses to VSV-MARV vaccination in NHPs, blood samples were collected weekly after i.m. vaccination with 1 × 10^7^ plaque-forming-units (pfu) (Figure 1A). No significant differences in the frequencies of CD4 T, CD8 T, or CD20 B cells were detected throughout the vaccination phase (Figures S1A–C). Induction of the adaptive immune response was measured by assessing T and B cell proliferation longitudinally. Since naïve T cells undergo a proliferative burst and differentiate into either central memory (CM) or effector memory (EM) T cells following antigen encounter, we measured changes in expression of Ki67 within these subsets as previously described (22). This analysis showed that proliferation within CD4 and CD8 T cell memory subsets peaked 7 DPV (Figures 1B,C). B cell proliferation within isotype switched memory and marginal-zone like (MZ-like) subsets peaked 14 DPV (Figure 1D). Although this increase was not statistically significant, it correlates with the detection of MARV GP-specific IgG which peaked 21 DPV (Figure 1E). We also attempted to determine the frequency of MARV GP-specific T cells using IFNγ capture ELISPOT, but in most animals the frequency of responding T cells was very low (Figure S1D).

### Transcriptional Profiling of the Immune Response to VSV-MARV Vaccination Reveals Strong Interferon Stimulated Gene Expression

**Figure 2:**
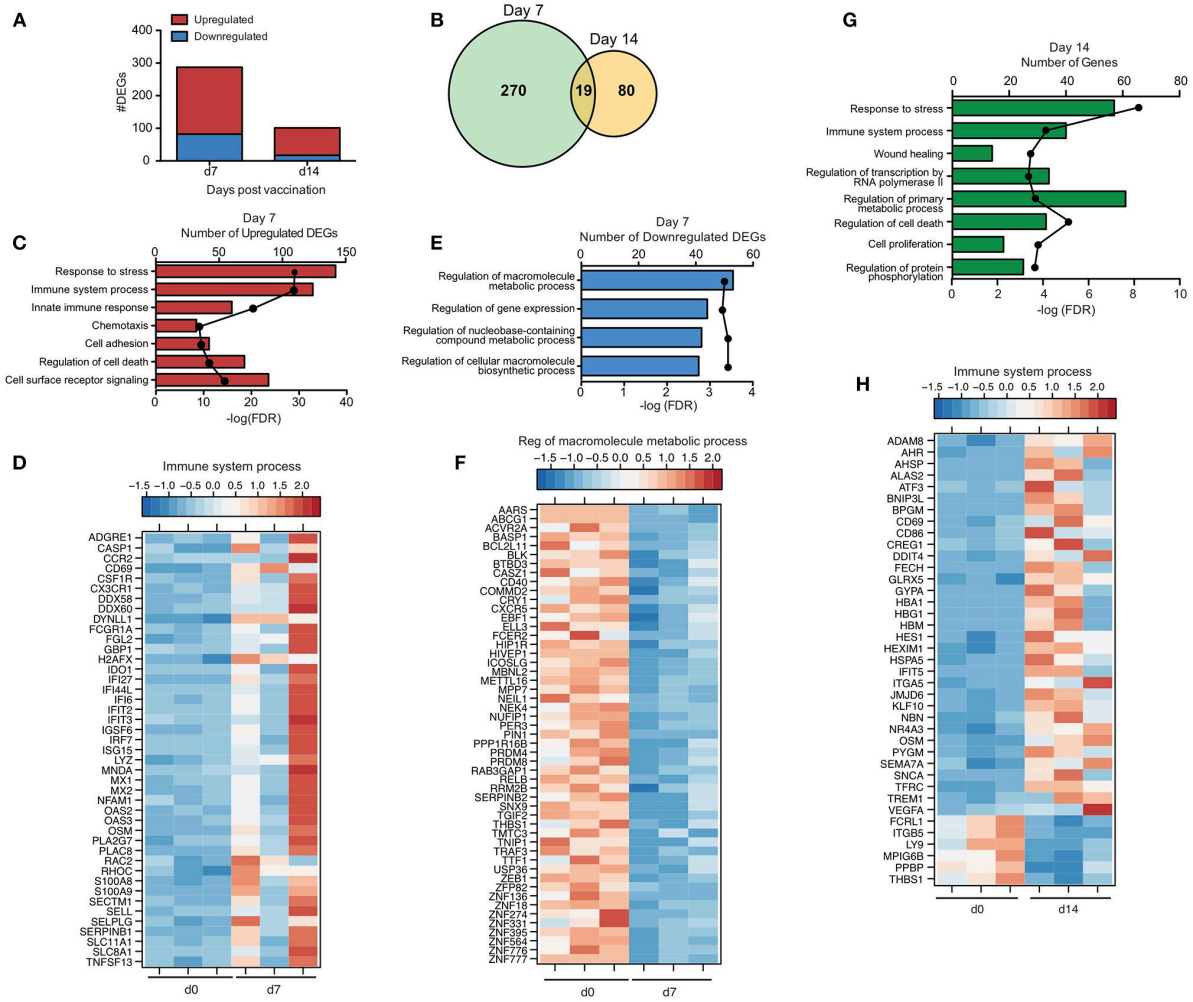
Transcriptional profile of the immune response to VSV-MARV vaccination. **(A)** Bar graph of differentially expressed protein coding genes (DEGs) detected 7 and 14 DPV [defined as those ≥ 2-fold change and false discovery rate (FDR) corrected *p* ≤ 0.05] that have human homologs (*n* = 3). **(B)** Venn diagram of DEGs detected 7 and 14 DPV in vaccinated animals. **(C)** Functional enrichment of genes upregulated 7 DPV; horizontal bar graphs represent number of genes while line graph represents FDR-corrected *p*-value. **(D)** Heatmap representing gene expression (shown as absolute normalized RPKM values) of upregulated DEGs with a FC >3 detected 7 DPV that enriched to “Immune system process”; range of colors is based on scaled and centered RPKM values of the entire set of genes (red represents increased expression while blue represents decreased expression); each column represents 1 animal. **(E)** Functional enrichment of downregulated genes at 7 DPV. **(F)** Heatmap representing gene expression of downregulated DEGs detected 7 DPV that enriched to “Regulation of macromolecule metabolic process”; each column represents 1 animal. **(G)** Functional enrichment of DEGs detected at 14 DPV. **(H)** Heatmap representing gene expression of DEGs detected 14 DPV that enriched to “Immune system process”; each column represents 1 animal.

To gain a better understanding of the host response to VSV-MARV vaccination, we measured changes in gene expression in PBMC samples collected 7 and 14 DPV (Figure 1A). Unsupervised clustering of differentially expressed genes (DEGs) following vaccination identified groups of genes that were co-regulated in a temporal manner, with transcriptional changes 7 DPV clustering further away from the day of vaccination and DPV 14 samples clustering closer to those observed DPV 0 indicative of a return to baseline (Figure S1E). Accordingly, the largest number of differentially expressed genes (DEGs) relative to pre-vaccination were detected at 7 DPV (289 DEGs) before declining on 14 DPV (99 DEGs) (Figure 2A). No differentially expressed genes were detected 21 DPV. Not surprisingly, given the outbred genetic background of the non-human primates used in this study, we observed some heterogeneity in the magnitude of gene expression changes observed (Figure S1E). For instance, although all gene expression changes were significant, animal 3 generated a more robust ISG response (cluster 1; Figures S1E,F), and animals 1 and 2 generated a higher fold change in the expression of other ISG/innate immune genes (cluster 2; Figures S1E,F). DEGs detected 7 and 14 DPV showed limited overlap (Figure 2B) with 19 DEGs that play a role in coagulation (e.g., *THBS1* and *F13A1*); immunity (*IFIT5, LY9*, and *CD69*); regulation of gene expression (e.g., *CREG1* and *KLF10*); and cell cycle regulation (e.g., *CDKN1A* and *OSM*). To infer the biological significance of the gene expression changes, we performed functional enrichment using MetaCore^TM^. This analysis revealed that DEGs upregulated at 7 DPV mapped to Gene Ontology (GO) terms associated with stress, immunity, signaling, and cell death (Figure 2C). A significant number of the upregulated DEGs at 7 DPV that mapped to “Immune system process” consisted of interferon stimulated genes (ISGs) that play a role in antiviral defense, notably *IRF7, IFIT2-3, MX1, OAS2*, and *DDX60* (Figure 2D). Other DEGs that enriched to this GO term play a role in inflammation (e.g., *S100A8 and 9, MNDA, CSF1R*, and *CCR2*); chemotaxis (e.g., *CX3CR1, DYNLL1, SELL*, and *SELPLG*); and apoptosis (e.g., *CASP1* and *TNFSF13*) (Figure 2D). Finally, we observed signatures of adaptive immunity such as increased transcripts of *CD69*, which is expressed on activated lymphocytes and *NFAM1*, which regulates B-cell signaling (Figure 2D). The small number of downregulated DEGs detected at day 7 enriched to GO terms associated with metabolism and gene expression (Figure 2E) and included: transcription factors *PRDM4, PRDM8, ELL3*; methyl transferase *METTL16*; and zinc finger proteins e.g., *ZNF18, ZNF274* (Figure 2F). Other downregulated DEGs play a role in inflammation and chemotaxis such as *RELB, TRAF3*, and *CXCR5*; and coagulation e.g., *SERPINB2* and *THBS1* (Figure 2F).

DEGs detected at 14 DPV mapped to GO terms associated with stress, cell death, transcription, cellular metabolism, and immunity (Figure 2G). Many upregulated DEGs that enriched to “Immune system process” play a role in red blood cell function including: hemoglobin subunits *HBA1* and *HBG1*; heme biosynthesis gene *ALAS2*; and iron procurement genes *TFRC, FECH* and *GLRX5* (Figure 2H). Expression of some innate immune genes including *IFIT5, TREM1* as well as *CD86* also increased (Figure 2H). The third major group of upregulated DEGs is critical to cell division and encompasses: transcription factors such as *ATF3* and *AHR*; transcription regulators including *KLF10, HEXIM1*, and *CREG1*; and cell cycle regulators e.g., *BNIP3L, OSM*, and *NBN* (Figure 2H). Notably, DEGs downregulated at 14 DPV included coagulation factors *THBS1* and *MPIG6B*, and neutrophil chemoattractant *PPBP* (Figure 2H).

To gain insight into the immune cells from which these gene expression changes originate, we used the Immunological Genome Project Consortium (ImmGen) database, which is a collaborative effort to delineate gene expression patterns across different immune cell populations (23). This analysis revealed that the source of the vaccine-induced DEGs is likely antigen presenting cells (DCs, monocytes and macrophages) with smaller contributions from B cells (Figures S2A,B).

### VSV-MARV Vaccination Is Highly Efficacious

**Figure 3:**
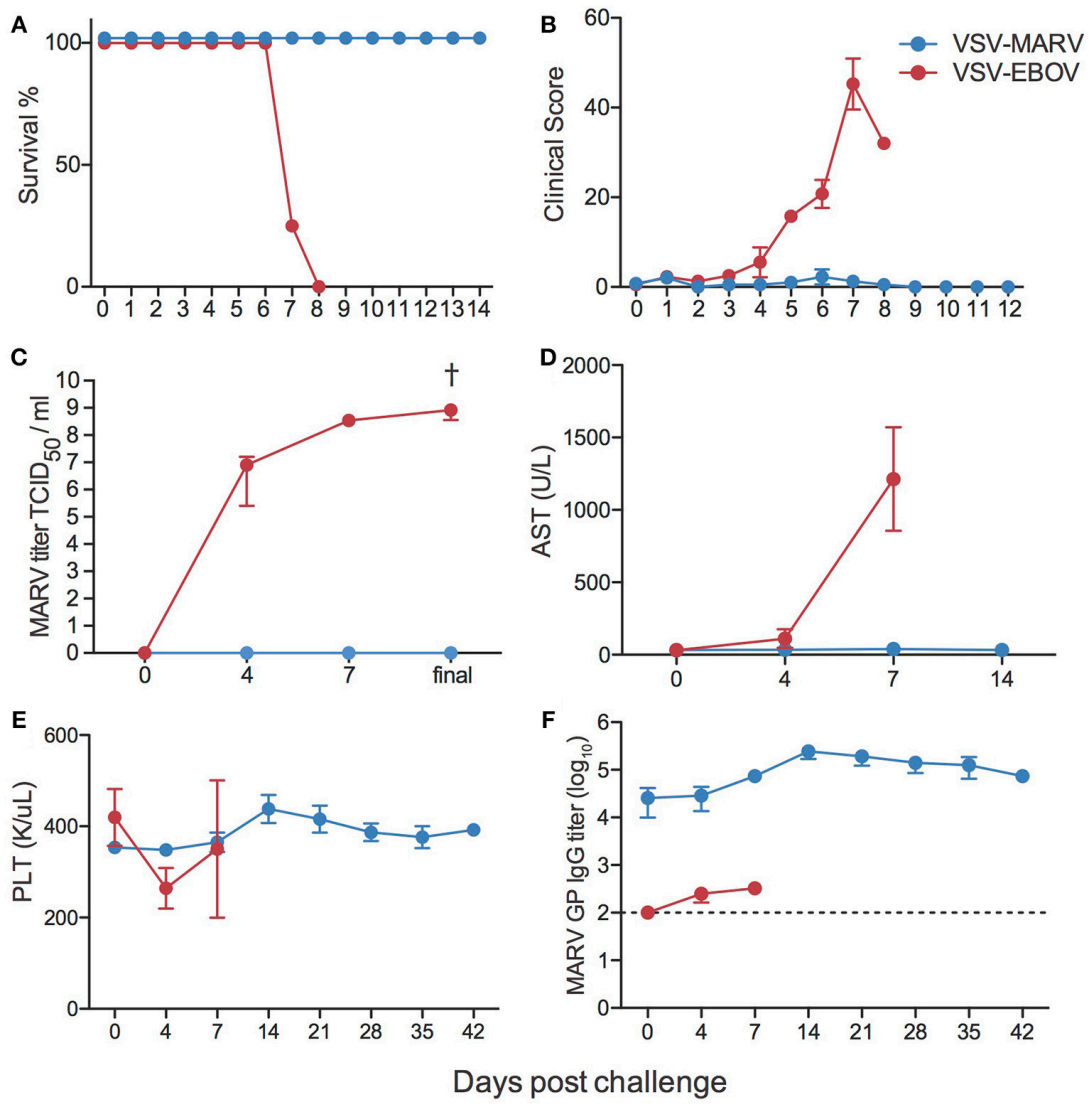
Clinical outcomes following MARV challenge. **(A)** Kaplan-Meier survival curves of negative control VSV-EBOV vaccinated (red) and VSV-MARV vaccinated (blue) animals (VSV-EBOV *n* = 4; VSV-MARV *n* = 6). **(B)** Average clinical scores as obtained by an approved scoring sheet during the challenge phase. **(C)** Average MARV titers from whole blood samples for the animals as determined on Vero E6 cells as 50% tissue culture infectious dose (TCID_50_). **(D)** Liver enzyme aspartate aminotransferase (AST) levels. **(E)** Platelet counts. **(F)** MARV GP-specific IgG endpoint titers were measured by ELISA; line graph represents limit of detection. Longitudinal changes were assessed using one-way repeated measures ANOVA test followed by Dunnett's multiple comparison post-test to determine differences between the time of challenge (day 0) and subsequent DPC, ^†^denotes VSV-EBOV animals; ^†^*p* < 0.05.

Animals were challenged i.m. with 1,000 pfu MARV 35 days following vaccination. As previously reported following vaccination with VSV-MARV expressing Musoke GP and challenge with MARV-Musoke (11, 13, 24–26), all animals vaccinated with VSV-MARV expressing Angola GP were completely protected and did not display signs of disease or develop viremia following challenge with MARV-Angola (Figures 3A–C). In contrast, animals that received the VSV-EBOV as a control vaccine succumbed 7–8 days post challenge (DPC) after reaching clinical scores requiring euthanasia and achieving high levels of viremia (Figures 3A–C). Consistent with hemorrhagic disease, negative control animals showed signs of liver damage as evidenced by increased levels of liver enzyme AST (Figure 3D), and several key cytokines such as IL-1β, IL-6, IL-1Ra, MIP-1α, TNFα, IFN-γ, IL-2, and FGF-β (Figure S3). In contrast to EBOV but in line with previous reports of MARV infection in NHPs (7), we did not observe a clinically relevant thrombocytopenia (Figure 3E). Interestingly, and in line with previous studies that reported an increase (27) in lymphocyte numbers, we detected an increase in white blood cell and lymphocyte numbers in the negative control animals, albeit not statistically significant, and a significant increase in lymphocytes in VSV-MARV vaccinated animals (Figure S4). Similar to previous studies, we measured an increase in MARV GP-specific IgG after challenge (Figure 3F) suggestive of a boost-effect on the immune response after MARV challenge.

**Figure 4:**
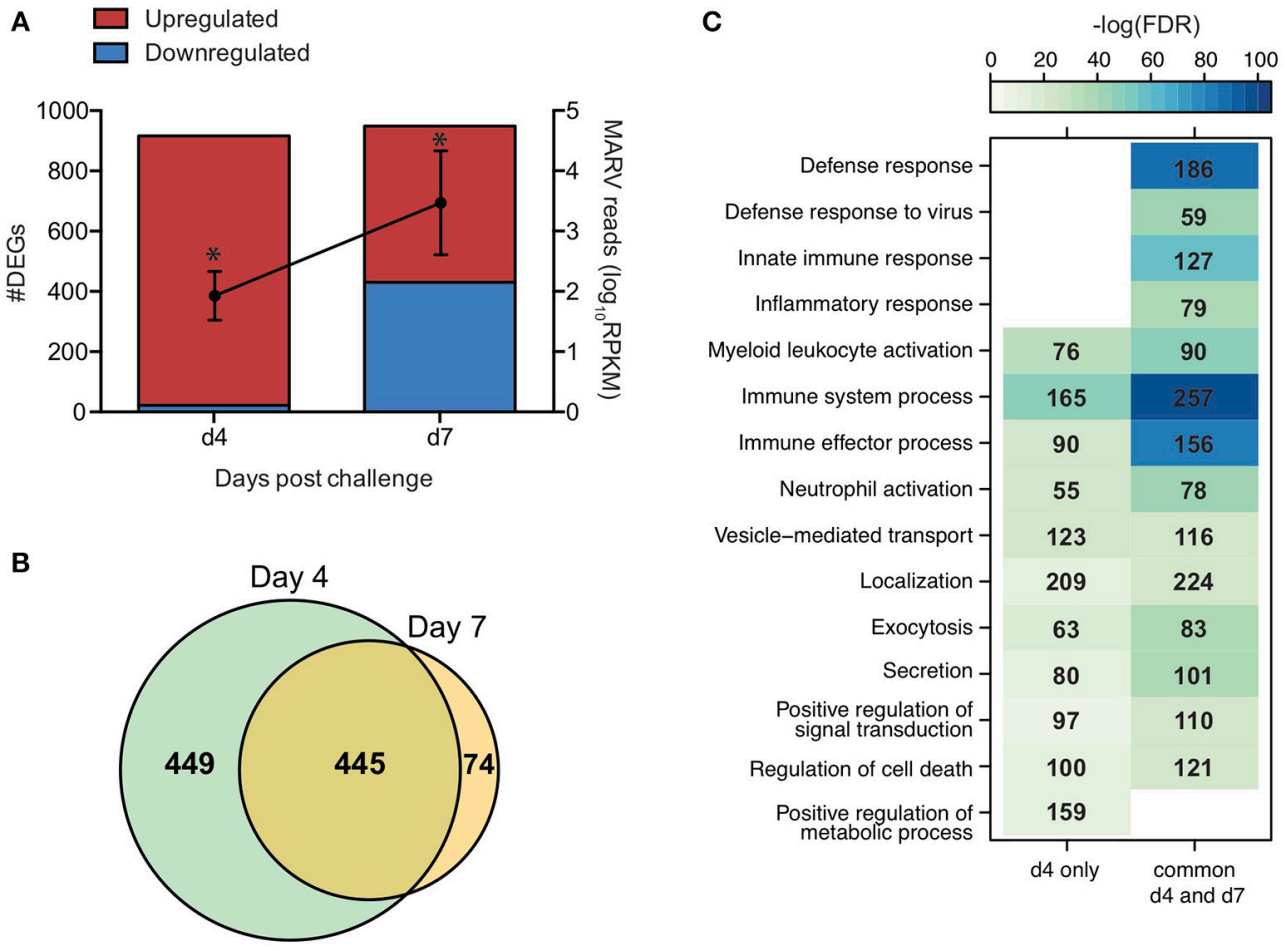
Gene expression changes following MARV challenge in negative control animals. **(A)** Bar graph depicts number of DEGs 4 and 7 DPC [defined as those ≥ 2-fold change and false discovery rate (FDR) corrected *p* ≤ 0.05] that have human homologs (*n* = 4). Line graph indicates number of viral transcripts reported as normalized by reads per kilobase per million mapped (RPKM); the *EdgeR* package was used to determine statistically significant changes in viral reads; ^*^denote *p* ≤ 0.05 at the indicated time point compared to 0 DPC. **(B)** Venn diagram of upregulated DEGs detected 4 and 7 DPC in negative control animals. **(C)** Heatmap representing functional enrichment of upregulated DEGs detected 4 DPC or 4 and 7 DPC; color intensity represents the statistical significance (shown as –log_10_ of the FDR-corrected *p*-value); range of colors is based on the lowest and highest –log_10_(FDR) values for the entire set of GO processes; the number of DEGs enriching to each GO process each day is listed within each box; blank boxes represent no statistical significance.

**Figure 5:**
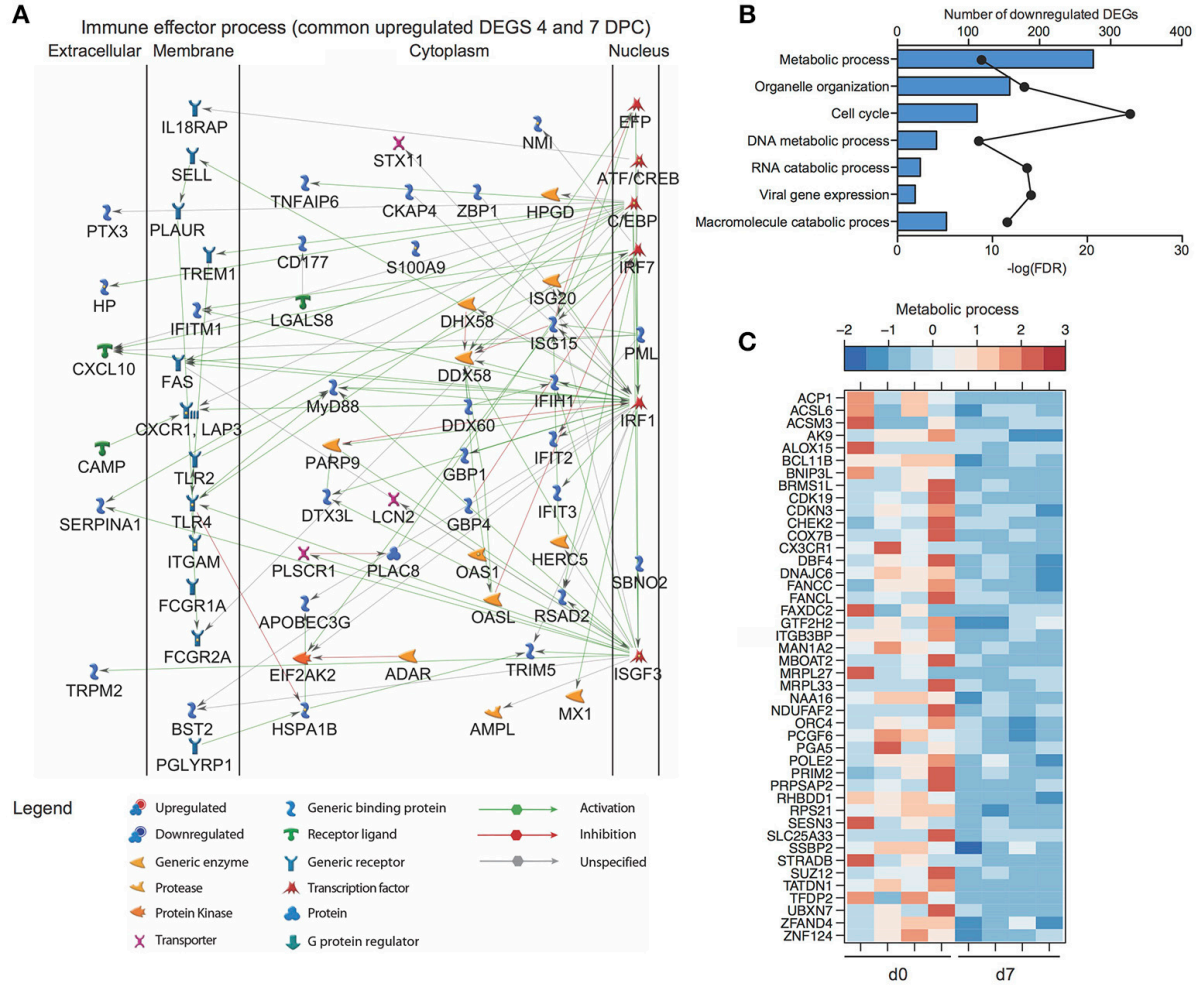
Transcriptional profiling of the host response to MARV challenge in negative control animals. **(A)** Network image of upregulated DEGs detected 4 and 7 DPC that enrich to “Immune effector process”. **(B)** Functional enrichment of downregulated genes 7 DPC in negative controls (*n* = 4); horizontal bar graphs represent number of genes that mapped to each GO term listed while line graph represents false discovery rate (FDR)-corrected *p*-value. **(C)** Heatmap representing gene expression (shown as absolute normalized RPKM values) of DEGs downregulated 7 DPC that enrich to the GO term “Metabolic process” (genes with fold change >5.4 are shown); range of colors is based on scaled and centered RPKM values of the entire set of genes (red represents increased expression while blue represents decreased expression); each column represents 1 animal.

In line with the lack of clinical symptoms and based on principal component analysis (Figure S5), we did not detect any DEGs in whole blood (WB) samples collected from VSV-MARV vaccinated protected animals. In contrast, a substantial number of DEGs was detected 4 (916 DEGs) and 7 DPC (956 DEGs) in WB from the negative control animals that received VSV-EBOV (Figure 4A). Approximately 449 upregulated DEGs were detected only 4 DPC (Figure 4B) and enriched to GO terms associated with immunity, secretion, signaling, cell death, and metabolism (Figure 4C). DEGs upregulated both 4 and 7 DPC enriched to similar GO processes as those described for DEGs detected 4 DPC only (Figure 4C). Several of the upregulated DEGs that were detected both 4 and 7 DPC and enriched to “Immune effector process” are regulated by transcription factors critical to an inflammatory response, notably: *ISGF3, IRF1* and *IRF7*, and *CEBP* (Figure 5A). DEGs in this work include ISGs (*IFIT1-3, OAS1-2, MX1*, and *ISG15*), inflammatory mediators (*PTX3, CXCL10, S100A8*, and *SERPINA1*), pathogen recognition receptors (*TLR2, TLR4*, and *MYD88)*, apoptosis (*CD95*), and genes that play a role in extracellular matrix degradation (*PLAUR* and *TNFAIP6*) (Figure 5A). The 75 DEGs upregulated only 7 DPC also play a role in host defense and inflammation (*CCL2, CD14, IGKC, IL2R2, MMP8, SIGLEC9*).

Downregulated DEGs were only detected 7 DPC and enriched to cell cycle, organelle organization, gene expression, and metabolic processes (Figure 5B). Decreased transcripts that enriched to “Metabolic process” included genes that play a role in the electron transport chain (*DNAJC6, COX7B*, and *NDUFAF2*), cell cycle (*CDKN3, CHEK2, DBF4*, and *PRIM2*), and DNA repair and replication (*POLE2, ORC4*, and *SSBP2*) (Figure 5C).

### Transcriptional Response to MARV Challenge Is Distinct From That Generated Following EBOV Challenge

**Figure 6:**
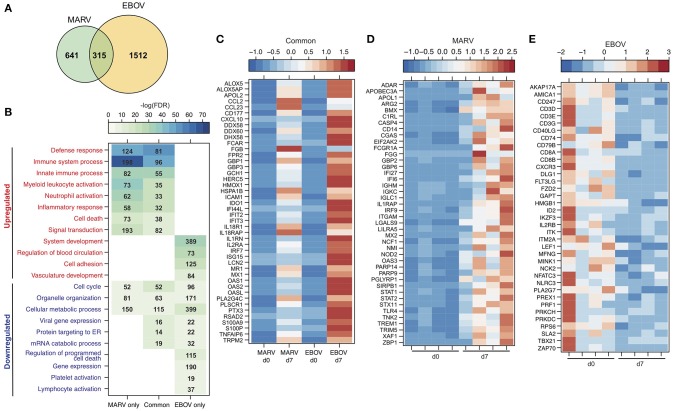
Transcriptional response to MARV infection indicates lesser disease severity compared to EBOV. **(A)** Venn diagram displaying overlap between DEGs detected following MARV or EBOV challenge 7 DPC. **(B)** Heatmap representing functional enrichment of DEGs detected following MARV or EBOV challenge 7 DPC; color intensity represents the statistical significance [shown as –log_10_ of the false discovery rate (FDR)-corrected *p*-value]; range of colors is based on the lowest and highest –log_10_(FDR) values for the entire set of GO processes; the number of DEGs enriching to each GO process each day is listed within each box; blank boxes indicate no significant enrichment to a GO term. **(C–E)** Heatmap representing gene expression (shown as absolute normalized RPKM values) of DEGs detected 7 DPC: **(C)** following either MARV or EBOV that enrich to “Defense response” (with a fold change > 11); **(D)** upregulated DEGs detected only following MARV infection that enrich to “Innate immune response” (with a fold change > 4.0); **(E)** downregulated DEGs detected only following EBOV infection that enrich to “Lymphocyte activation”. Range of colors is based on scaled and centered RPKM values of the entire set of genes (red represents increased expression while blue represents decreased expression); each column represents the median RPKM values on each day for either group in **(C)** while each column represents 1 animal in **(D,E)**.

To better understand the pathogenesis caused by MARV, we compared the transcriptional profile detected in the negative controls of this study to a previous study in which the negative control animals were vaccinated with VSV-MARV and challenged with 1,000 pfu EBOV-Kikwit (22, 28). Library preparation, sequencing, and bioinformatics analyses were carried out in the same manner. More importantly, both cohorts were vaccinated with a recombinant VSV-based vaccine i.m. and challenged with the respective virus by the same route, making a direct comparison possible. A much larger number of DEGs was detected following EBOV infection compared to MARV (Figure 6A). Although there was significant overlap, we also identified transcriptional changes that were unique to each virus (Figure 6A). Functional enrichment showed that DEGs detected following infection with either MARV or EBOV enriched to GO terms related to host defense (innate immunity, myeloid cell activation and inflammatory processes), cell death, cell cycle and metabolism (Figure 6B). The majority of shared DEGs were ISGs including *IFIT2&3, ISG15, MX1*, and *OAS1&2* and inflammatory genes such as *CCL2, CXCL10, IL1RN*, and *S100A9* (Figure 6C).

DEGs found exclusively during MARV infection also enriched to GO terms related to host defense, inflammation, cell death and cell cycle (Figure 6B). Specifically, expression of genes involved in the complement response such as *C1RL*; neutrophil and monocyte recruitment *CD14, NCF*, and *TREM1*; and innate immune signaling e.g., *STAT1&2, IRF9*, and *TLR2* was increased for MARV-infections (Figures 6B,D). In contrast, DEGs found exclusively during EBOV infection were mostly involved with dysregulation of blood circulation and vasculature development as well as lymphopenia as evidenced by a decrease in *CD79B, CD3, CD8, NFATC3*, and *PRF1* (Figures 6B,E).

### Analysis at Late Stage of Disease Reveals Overlapping Yet Distinct Transcriptional Profile Compared to MARV Aerosol Challenged NHPs

**Figure 7:**
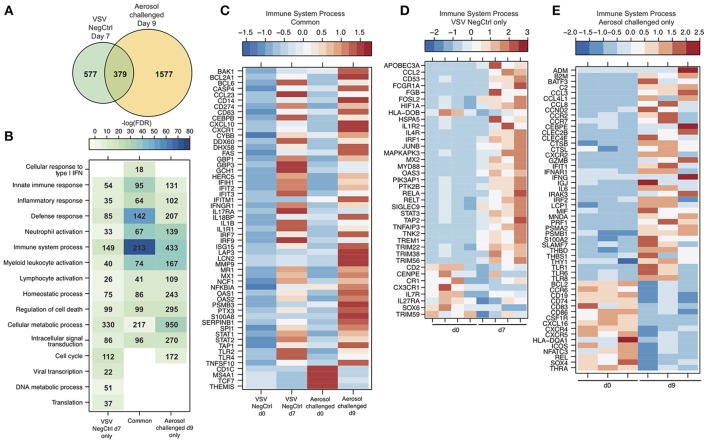
Analysis at late stage of disease reveals overlapping yet distinct transcriptional profile compared to MARV aerosol challenged NHPs. **(A)** Venn diagram displaying overlap between DEGs detected VSV-EBOV negative control animals at 7 DPC and MARV aerosol challenged animals at 9 DPC. **(B)** Heatmap representing functional enrichment of DEGs detected in each group; color intensity represents the statistical significance [shown as –log_10_ of the false discovery rate (FDR)-corrected *p*-value]; range of colors is based on the lowest and highest –log_10_(FDR) values for the entire set of GO processes; the number of DEGs enriching to each GO process each day is listed within each box; blank boxes represent no statistical significance. **(C–E)** Heatmap representing gene expression (shown as absolute normalized RPKM values) of a select subset of DEGs that enrich to “Immune system process” detected in both groups **(C)**, in VSV-EBOV negative control animals only **(D)**, and MARV aerosol challenged animals **(E)**. Range of colors is based on scaled and centered RPKM values of the entire set of genes (red represents increased expression while blue represents decreased expression). Range of colors is based on scaled and centered RPKM values of the entire set of genes (red represents increased expression while blue represents decreased expression); each column represents the median RPKM values on each day for either group in **(C)** while each column represents 1 animal in **(D,E)**.

We compared our gene expression changes detected in negative control cynomolgus macaques to those reported following aerosol MARV-Angola challenge in rhesus macaques (29). Due to the macaque species and the route of exposure, animals succumbed 1–2 days later than in our study. Therefore, we compared DEGs detected in our negative control animals and aerosol MARV challenged animals at 7 and 9 DPC, respectively. DEGs detected at end stage of disease for both groups showed significant overlap as well as unique transcriptional changes (Figure 7A). Interestingly, most of the DEGs regardless of whether they were common or unique to the challenge route enriched to similar GO terms (Figure 7B). We further investigated DEGs that mapped to “Immune system process.” As expected, common DEGs included ISGs e.g., *IFIT2&3, ISG15, MX1*, and *OAS2*; genes involved in inflammation including *CXCR1, CD14, CXCL10, IL1B*, and *S100A8*; antigen presentation including *TAP1, CD1C, and MR1*; apoptosis such as *BAK1, CASP4, TNSF10*, and *FAS*; and T-cell inhibition such as *CD274* (Figure 7C). Transcriptional changes that were only detected in this study included genes important for TLR signaling (*MYD88, RELA*, and *STAT3*); regulation of cell proliferation such as *JUN, PTK2B* and *TNK2*; and lymphocyte related transcripts (e.g., *CD2, IL7R*, and *IL27RA*) which were downregulated (Figure 7D). DEGs that were uniquely identified in aerosol MARV challenged animals included genes important for inflammation (*IFNG, IL6, MIF*, and *S100A2*); chemotaxis (*CCL3, CCR2, CCL8*, and *CCR7*); antigen presentation and co-stimulation (*HLA-DQA1, CD74, CD83*, and *CD86*); and adaptive immunity (*CD19, CXCR5, CCR6*, and *NFATC3*) (Figure 7E).

## Discussion

Previous studies have shown that VSV vectors expressing either EBOV or MARV GPs are highly efficacious against lethal filovirus challenge in preclinical studies including cynomolgus and rhesus macaque models (9). More importantly, the VSV-EBOV vaccine is the only one to successfully complete a phase III clinical trial during the West African EBOV epidemic (16, 30). Recent studies using NHPs revealed that antibodies play a critical role in protection conferred by VSV-EBOV (22). Additional studies reported that VSV-EBOV engenders a robust innate immune response and that CD8 T cells play a role, albeit limited in protection (28). However, no studies to date have investigated the host response to VSV-MARV vaccination or the transcriptional response to MARV infection following intramuscular injection. In this study, we report the first longitudinal transcriptional analysis following VSV-MARV vaccination and subsequent MARV challenge in protected survivors and negative control animals. We utilized the updated VSV-MARV vaccine expressing the MARV-Angola GP instead of the original MARV-Musoke GP which resulted in increased replication kinetics *in vitro* (18). This is the first NHP study demonstrating 100% protective efficacy of this updated VSV-MARV vaccine in NHPs, the gold standard model for MARV vaccine efficacy studies. Similar to the VSV-MARV-Musoke vaccine, we achieved complete protection with a single vaccine dose administered 35 days prior to lethal challenge.

Our data revealed that VSV-MARV vaccination induced gene expression changes associated with innate immunity 7 and 14 DPV. Some variation in the magnitude of the immune response was observed between the three animals following vaccination most likely due to the use of an outbred species and potentially differences in kinetics of the response within each animal. Nevertheless, we observed an upregulation of ISGs important for antiviral defense in addition to genes that are critical to neutrophil and monocyte responses in all the animals. We also detected increased transcripts important for T cell activation and B cell signaling. These transcriptional changes correlated with T and B cell proliferation which peaked 7 and 14 DPV and MARV GP-specific IgG peaking at 21 DPV. Additional *in silico* analyses also predict that the majority of these gene expression changes originate primarily from antigen presenting cells with smaller contributions from B and T cells. Future experiments will leverage novel single cells genomic technologies to better understand the contributions of specific immune population to the vaccine. Previous studies revealed that Type I IFNs play important roles in the generation of Tfh cells and that pDCs are critical for the generation of plasma cells and anti-viral antibodies from B cells via type I IFN signaling. Therefore, the increased expression of ISGs detected 7 and 14 DPV may promote the development of humoral responses. It is intriguing that antibody titers are detected despite a modest proliferative burst in peripheral B cells. This observation suggest that the bulk of the B cell proliferation may be occurring in draining lymph nodes, which were not sampled in the current study. Interestingly, increased expression of erythropoiesis related genes was detected 14 DPV. Although humoral response increased in vaccinated animals following MARV challenge, we did not detect gene expression changes due to the fact that antibody affinity maturation occurs in secondary lymphoid tissue such as lymph nodes and spleen rather than blood. Therefore, future studies should focus on delineating host immune responses following both vaccination and challenge within secondary lymphoid tissues and within antigen-specific immune cell subsets that can be enriched using labeled GP for instance in the case of B cells.

We recently determined the transcriptional changes induced by VSV-EBOV expressing EBOV-Mayinga GP (28). A comparison of these two data sets showed that VSV-MARV expressing Angola GP induced larger gene expression changes that were sustained over a longer duration of time. Specifically, while VSV-EBOV vaccination altered the expression of only 60 DEGs 7 DPV, VSV-MARV resulted in changes in 369 genes at 7 and 14 DPV. Surprisingly, only 10 DEGs were shared between the two data sets (*ARL6IP5, GBP6, FGD2, HIST1H1B, IFI44L, IFI44, IFIT2, IFIT3, OAS2*, and *PLAC8*). However, despite the limited overlap, both vaccines elicit a strong innate immune response, dominated by type I IFN signaling, which may direct B cell activation and antibody production. Neither vaccine engenders a measurable T cell response post vaccination against GP. These data suggest that MARV-Angola GP may be a more immunogenic antigen than EBOV-Mayinga GP. Indeed, T cell proliferation was detected 7 days post VSV-MARV vaccination compared to 14 days post VSV-EBOV vaccination (22). While both GPs have a similar trimeric structure on the VSV surface, the amino acid sequence and the glycosylation patterns of the proteins differ substantially which may impact immunogenicity (6, 31). A higher immunogenicity of the MARV GP would be consistent with the complete absence of MARV reads or transcriptional changes in the VSV-MARV vaccinated protected animals following lethal MARV challenge. In contrast, limited viral and host gene expression changes were detected in VSV-EBOV vaccinated protected animals (28, 32, 33).

Gene expression changes in unprotected animals were consistent with the clinical features of MHF. Genes upregulated throughout infection mostly consisted of ISGs, pro-inflammatory genes, and those important for cell death similar to transcriptional changes following EBOV infection (33, 34). Furthermore, the largest number of upregulated DEGs associated with cell death and inflammation was detected 4 DPC, which preceded the significant increase of inflammatory mediators 7 DPC. We also detected a downregulation of genes associated with cell cycle, gene expression and metabolism 7 DPC. These decreased transcripts suggest either cell death and/or efforts to prevent further viral replication and spread. In contrast to EBOV, we detected a decrease in very few lymphocyte related transcripts, which correlated with the relatively stable number of lymphocytes following MARV challenge. Additionally, gene expression profiles did not indicate abnormalities in hemostasis or vasculature development, consistent with the lack of clinically significant thrombocytopenia. However, upregulated DEGs detected exclusively following MARV challenge suggest more significant dysregulation of innate immunity and cell death. Therefore, while both MARV and EBOV infection result in multi-organ failure and septic shock, these data suggest that some aspects of MARV pathogenesis are different from EBOV infection.

Compared to aerosol MARV-Angola challenged rhesus macaques, we identified similar gene expression signatures of dysregulated inflammation, however, it was much more pronounced in the aerosol challenged animals potentially due to the protracted disease course. Several additional factors could explain differences between the two studies, notably the Connor et al., study used PBMC and high-density micro-arrays while we used WB samples and RNA-Seq as well as differences between host responses of rhesus macaques used in the Connor et al., study vs. cynomolgus macaques used in this study.

In summary, our findings demonstrate that VSV-MARV-Angola induces strong innate responses that can direct the development of protective humoral responses. In addition, the lack of any gene expression changes or viral reads in VSV-MARV-Angola vaccinated and protected animals following MARV challenge highlight that the VSV-MARV-Angola is a highly effective vaccine.

## Author Contributions

AM, HF, and IM conceived and designed the study. AM, FF, and HF performed the animal study. AM, ARM, FE, JC, EJH, and IM processed the samples. AM, ARM, AJ, HF, and IM analyzed the data and wrote the manuscript. All authors approved the manuscript.

### Conflict of Interest Statement

HF claims intellectual property regarding vesicular stomatitis virus-based vaccines for Marburg virus infections. The remaining authors declare that the research was conducted in the absence of any commercial or financial relationships that could be construed as a potential conflict of interest. The reviewer JC declared a past supervisory role with one of the authors (AM) to the handling Editor.
