# Supplementary material for: Protection Against Marburg Virus Using a Recombinant VSV-Vaccine Depends on T and B Cell Activation
Source: Front Immunol. 2019 Jan 22;9:3071. doi: 10.3389/fimmu.2018.03071 (PMC6350103; doi:10.3389/fimmu.2018.03071)
Supplement: Supplementary file 1 [file Data_Sheet_1.pdf]

Protection against Marburg virus using a recombinant VSV-vaccine depends on T and B cell activation

Andrea Marzi[1]*, Andrea R. Menicucci[2], Flora Engelmann[3], Julie Callison[1], Eva J. Horne[1], Friederike Feldmann[4], Allen Jankeel[2], Heinz Feldmann[1], Ilhem Messaoudi[2]*

[1]Laboratory of Virology, Division of Intramural Research, National Institute of Allergy and Infectious Diseases, National Institutes of Health, Rocky Mountain Laboratories, Hamilton, MT 59840, USA
[2]Department of Molecular Biology and Biochemistry, University of California-Irvine, Irvine, CA 92697, USA
[3]Department of Cell Molecular Biology, Northwestern University, Evanston, IL 60208, USA
[4]Rocky Mountain Veterinary Branch, Division of Intramural Research, National Institute of Allergy and Infectious Diseases, National Institutes of Health, Hamilton, MT 59840, USA

Supplementary figures

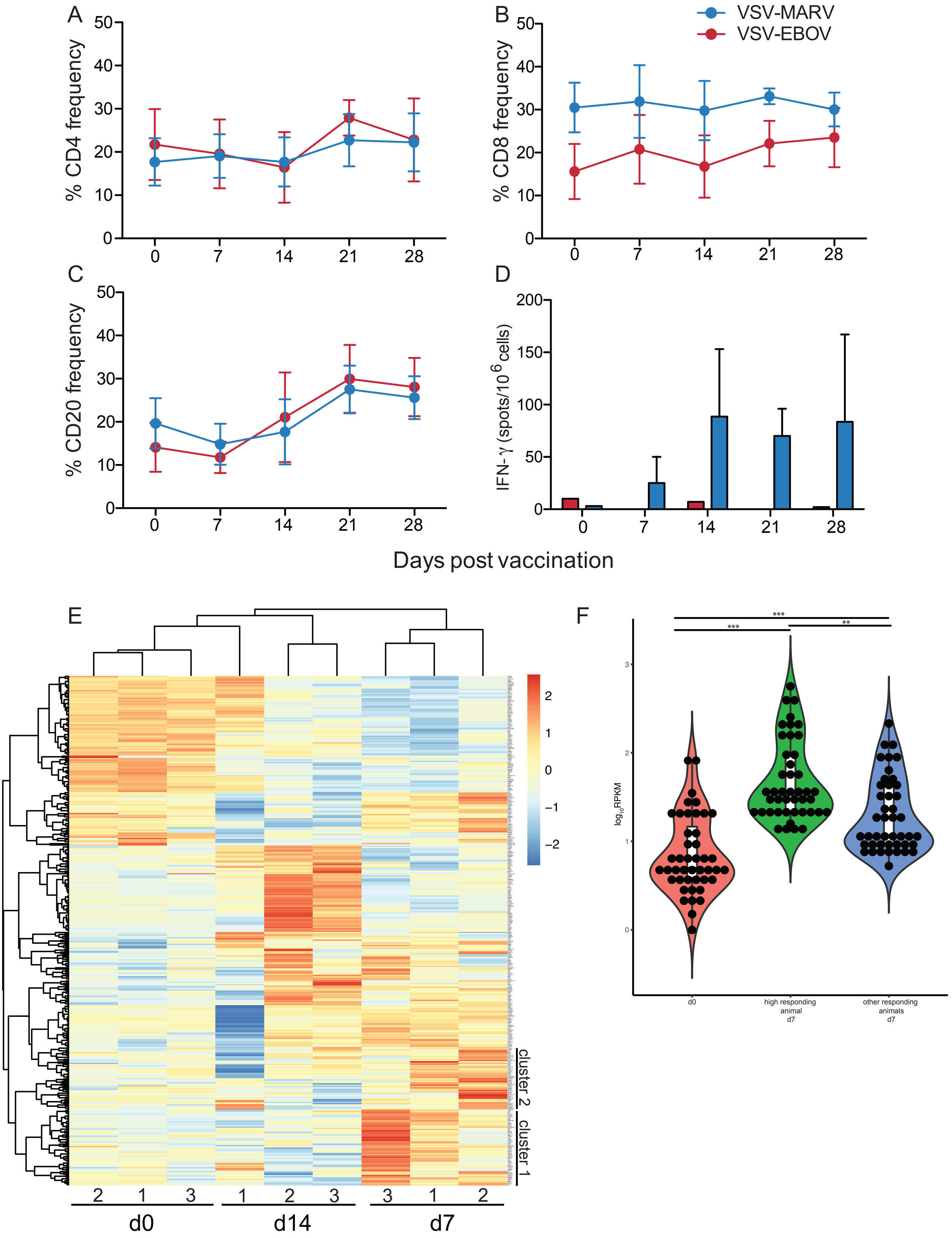

A
% CD4 frequency
B
% CD8 frequency
VSV-MARV
VSV-EBOV
C
% CD20 frequency
D
IFN-γ (spots/10^6 cells)
0
7
14
21
28
Days post vaccination
E
2
1
0
−1
−2
cluster 2
cluster 1
2 1 3
1 2 3
3 1 2
d0
d14
d7
F
log10RPKM
***
***
**
d0
high responding animal d7
other responding animals d7

Figure S1. Lymphocyte frequency and MARV-specific T cell responses. Frequency of CD4 T cells (A), CD8 T cells (B) and CD20 B cells (C) in VSV-MARV vaccinated (positive control) animals (blue; n=4) and VSV-EBOV vaccinated (negative control) animals (red; n=3). (D) Frequency of MARV-specific T cells was measured using ELISPOT technology following stimulation of PBMCs collected after vaccination with overlapping peptide library covering MARV GP. Longitudinal changes were assessed using one-way repeated measures ANOVA test followed by Dunnett's multiple comparison post-test to determine differences between the time of vaccination (day 0) and subsequent days post vaccination. (E) Heatmap representing hierarchical clustering of samples and genes that were differentially expressed at 7 and/or 14 DPV. Range of colors is based on rlog transformation of read counts (red represents increased expression while blue represents decreased expression); each column represents 1 animal. (F) Violin plot of the $\log_{10}$RPKM of DEGs 7 DPV that enriched to "immune system process" (as shown in Figure 2D) at 0 DPV, animal 3 at 7DPV (high responding animal), and animal 1 and 2 at 7 DPV (other responding animals).

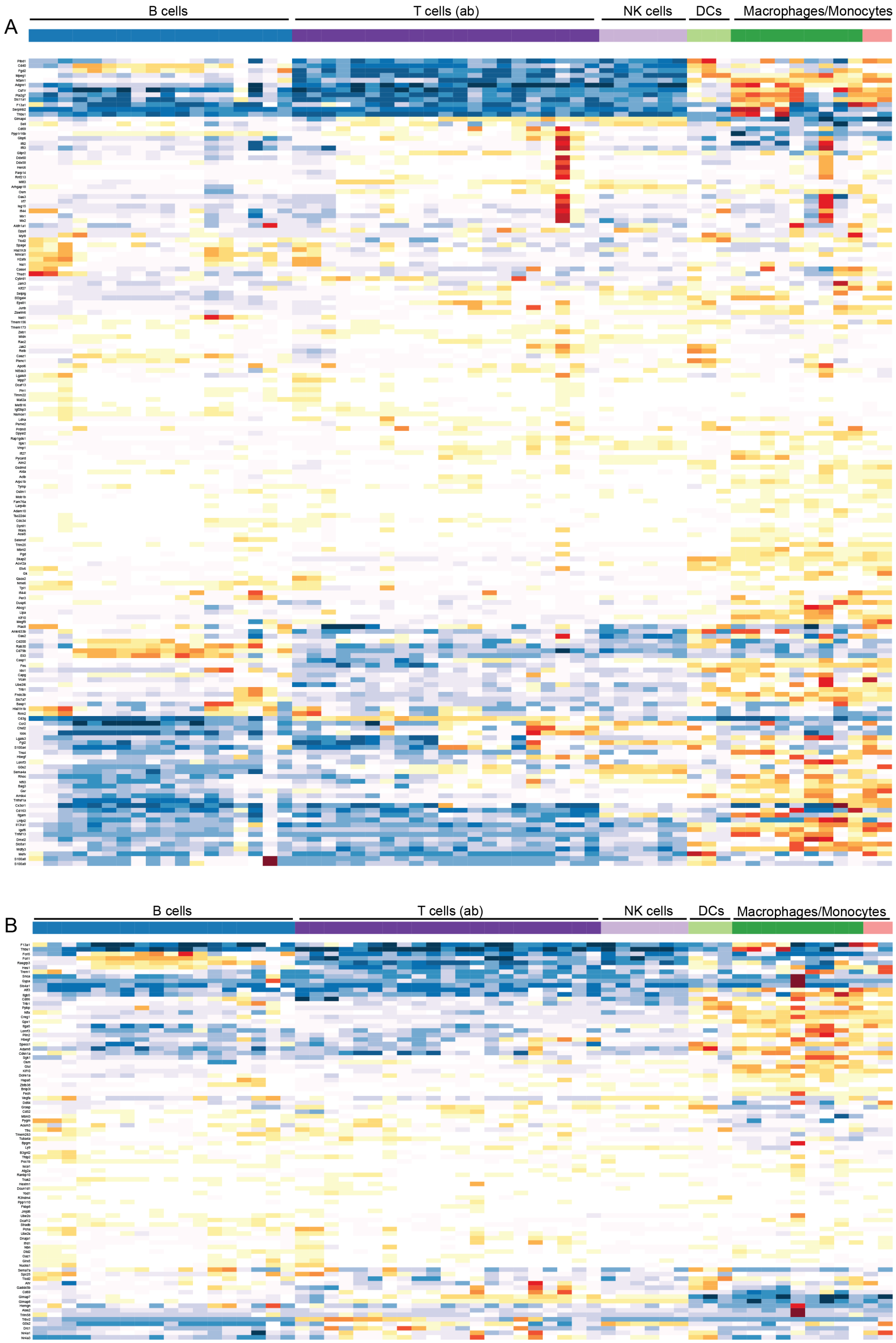

Figure S2. ImmGen analysis of PBMCs 7 and 14 days post VSV-MARV vaccination. Heatmap showing expression profile of DEGs, with fold change > 2.5, detected 7 DPV **(A)** and 14 DPV **(B)** across various immune cell populations as predicted by ImmGen's MyGeneSet application. Red indicates high while blue indicates low likelihood of expression within indicated immune cell population.

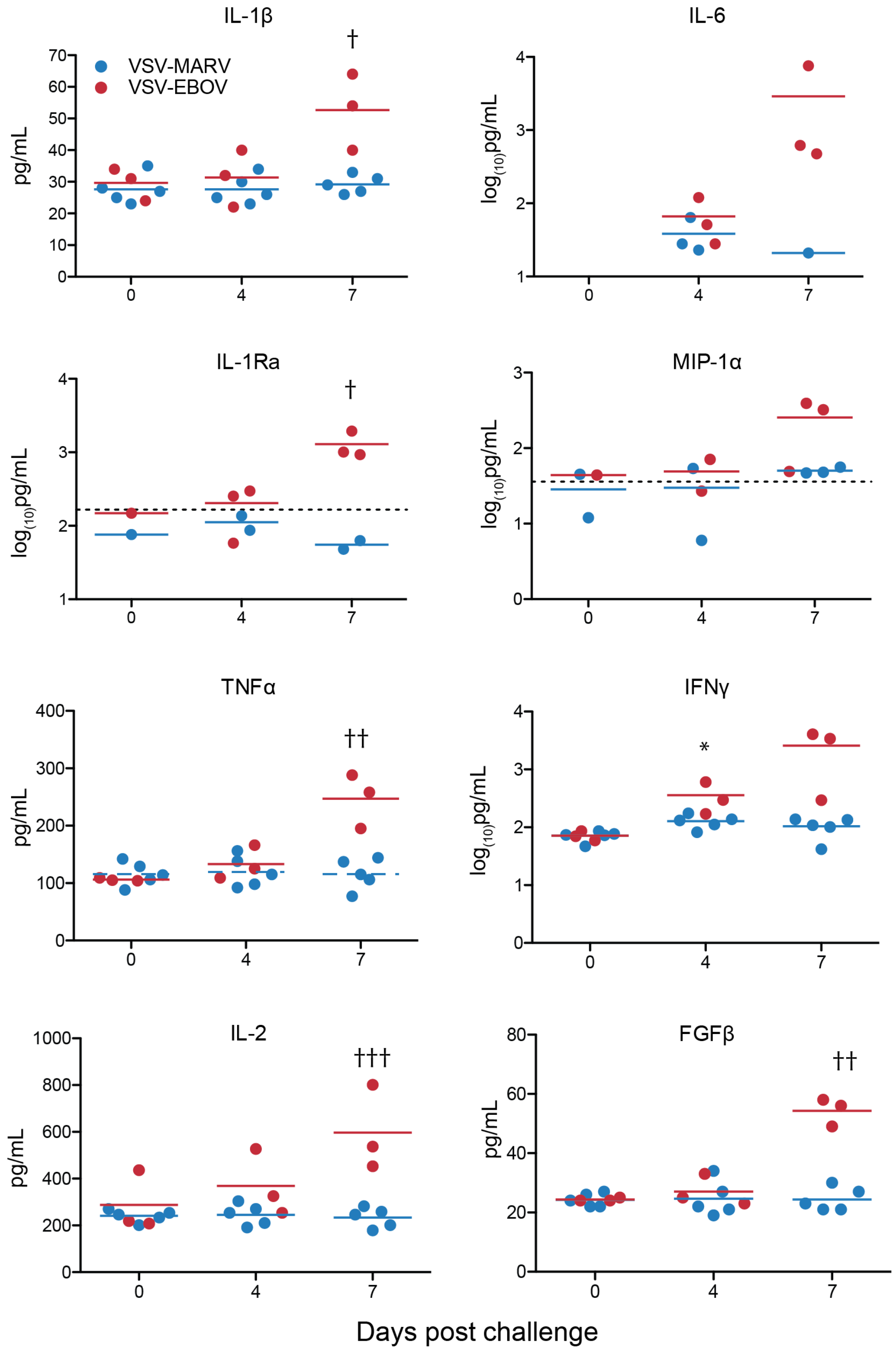

Figure S3. Plasma levels of cytokines, chemokines and growth factors. Plasma levels of IL-1β, IL-6, IL-1Ra, MIP-1α, TNFα, IFN-γ, IL-2, and FGF-β were measured using Luminex technology (VSV-EBOV n=3; VSV-MARV n=5). Longitudinal changes of cytokines were carried out using one-way repeated measures ANOVA test followed by Dunnett's multiple comparison post-test to determine differences between the time of challenge (day 0) and subsequent days post challenge; * denotes VSV-MARV animals, † denotes VSV-EBOV animals; * or † $p < 0.05$; †† $p< 0.01$; ††† $p < 0.001$.

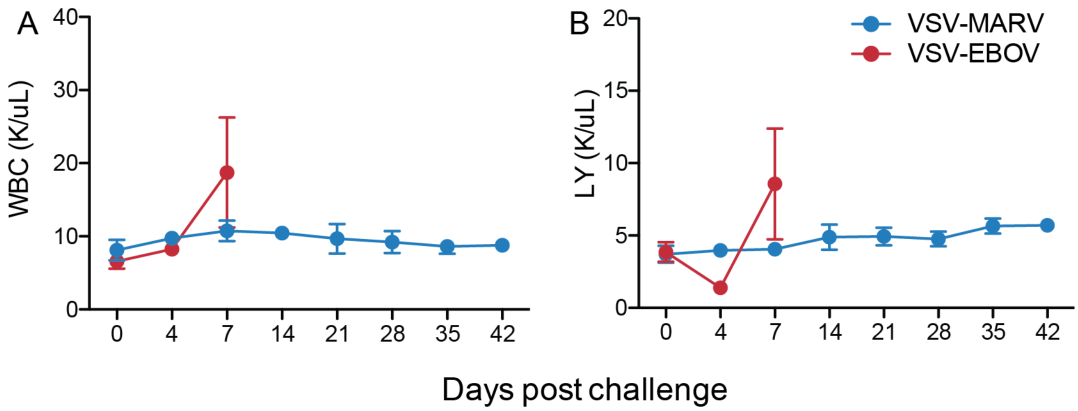

Figure S4. Blood cell counts following challenge. (A, B) Counts of White blood cell (WBC) (VSV-EBOV n=4; VSV-MARV n=6) **(A)** and lymphocytes (LY) (VSV-EBOV n=4; VSV-MARV n=6) **(B)** throughout infection.

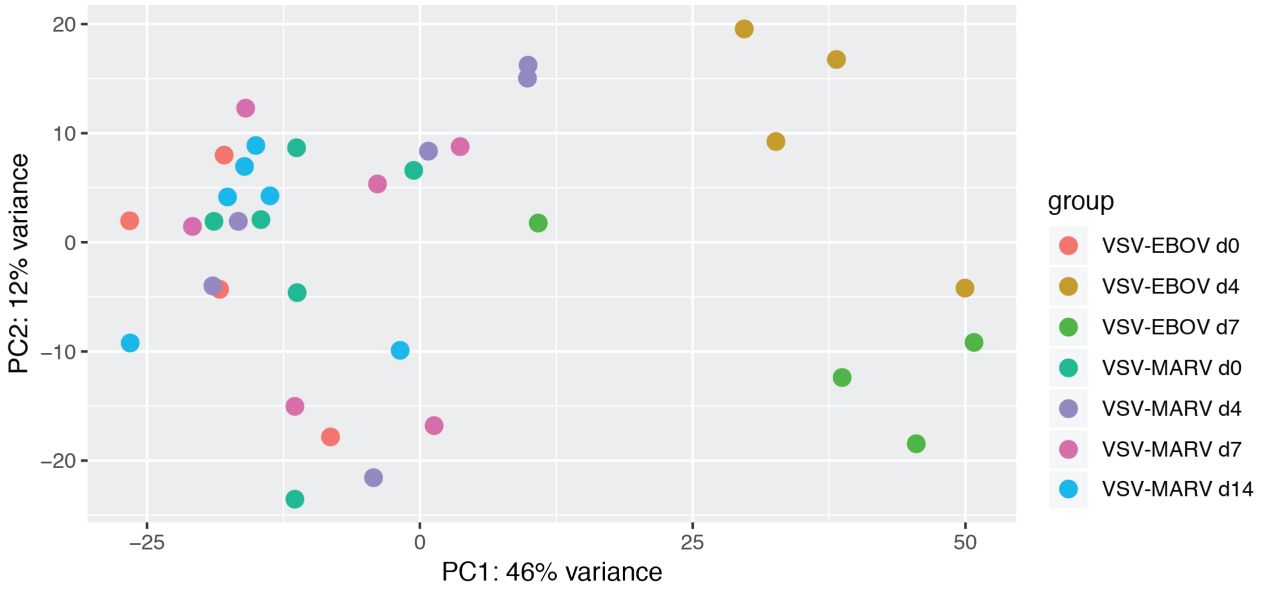

Figure S5. Principal component analysis post challenge. Principal component analysis of normalized transcript numbers in negative control animals and VSV-MARV vaccinated animals following MARV challenge. Each dot represents 1 biological replicate.
